# What evidence exists on the effects of public policy interventions for achieving environmentally sustainable food consumption? A systematic map protocol

**DOI:** 10.1186/s13750-022-00271-1

**Published:** 2022-04-25

**Authors:** Biljana Macura, Ylva Ran, U. Martin Persson, Assem Abu Hatab, Malin Jonell, Therese Lindahl, Elin Röös

**Affiliations:** 1 Stockholm Environment Institute, P.O. Box 24218, 104 51 Stockholm, Sweden; 2 Department of Energy and Technology, Swedish University of Agricultural Sciences, 750 07 Uppsala, Sweden; 3 Physical Resource Theory, Department of Space, Earth & Environment, Chalmers University of Technology, 412 96 Göteborg, Sweden; 4 Department of Economics, Swedish University of Agricultural Sciences, 750 07 Uppsala, Sweden; 5 Beijer Institute of Ecological Economics, The Royal Swedish Academy of Sciences, 104 05 Stockholm, Sweden; 6 Stockholm Resilience Centre, Stockholm University, Stockholm, Sweden; 7 The Royal Swedish Academy of Sciences, Stockholm, Sweden; 8 Nordic Africa Institute, SE-751 47 Uppsala, Sweden

**Keywords:** Biodiversity loss, Climate change, Environmental impacts, Greenhouse gas emissions, Public policy, Sustainable consumption, Sustainable diets

## Abstract

**Background:**

The global food system is causing considerable environmental harm. A transition towards more sustainable consumption is needed. Targeted public policy interventions are crucial for stimulating such transition. While there is extensive research about the promotion of more environmentally sustainable food consumption, this knowledge is scattered across different sources. This systematic map aims to collate and describe the available evidence on public policy interventions such as laws, directives, taxes and information campaigns, for achieving sustainable food consumption patterns.

**Methods:**

We will search bibliographic databases, specialist websites, Google Scholar and bibliographies of relevant reviews. Searches for academic literature will be performed in English, while searches for grey literature will be performed in English, Swedish, Danish and Norwegian. Screening, including consistency checking exercises, will be done at two levels: title and abstract, and full text. We will use machine learning algorithms to support screening at the title and abstract level. Coding and meta-data extraction will include bibliographic information, policy details and context, and measured environmental outcome(s). The evidence base will be summarised narratively using tables and graphs and presented as an online interactive searchable database and a website that will allow for visualisation, filtering and exploring systematic map findings, knowledge gaps and clusters.

**Supplementary Information:**

The online version contains supplementary material available at 10.1186/s13750-022-00271-1.

## Background

The global food system causes immense environmental impacts. Production of food is responsible for more than a third of greenhouse gas emissions [1], a third of terrestrial acidifications and almost 80% of eutrophication globally [2]. Further, food production requires large amounts of water [3] and land [2, 4]. Agricultural land conversion for food production is one of the largest contributors to biodiversity loss [5].

These production-related impacts are driven by food consumption patterns. Global demand for food, fuel and fibre, exacerbated by global population growth, urbanization and increasing affluence, is expected to keep putting pressure on the world’s ecosystems [2, 6]. Household-level consumption is associated with approximately 60% of global greenhouse gas emissions and food is amongst the most impactful consumption categories in terms of global greenhouse gas emissions [7, 8]. In Sweden, for example, greenhouse gas emissions from food consumption make up about a fourth of the total yearly household consumption-based emissions per person [9]. To stop further erosion of the global natural capital it is, thus, imperative to transform to a sustainable food system, including improvements in food consumption patterns. Sustainable food consumption can be defined as the use of goods and services that respond to basic needs and bring a better quality of life, while minimizing the use of natural resources, toxic materials and emissions of waste and pollutants over the life cycle, so as not to jeopardize the needs of future generations [10].

The transformation towards sustainable food consumption, however, requires action along the entire food supply chain and by a multitude of actors, including targeted policy interventions to modify purchasing decisions of retailers and end-consumers [11, 12]. Public policies (i.e., actions made by governments with specific goals and means (sensu [13, 14])) and governmental decision-makers [15] are key factors and actors in achieving sustainable transformation of the food systems [16, 17]. Moreover, policies and policy interventions designed to mitigate environmental impacts of consumption, for example, laws, taxes and information campaigns, and contribute to more sustainable food choices are receiving increased attention, both in research and politics [18–21], however, their effectiveness is largely unknown [22–24]. Moreover, many studies have investigated public policies and policy interventions applied by different levels of government to promote or incentivise sustainable food consumption. However, these studies are scattered across different sources and literature on the role of public policies in governing food consumption to reach food system sustainability targets, is insufficiently mapped.

### Policy interventions for sustainable food consumption

There are several theoretical approaches on consumer behaviour that can be used for investigating public policy interventions aiming to govern towards more sustainable food consumption [25, 26]. These include, for example, the theory of planned behaviour [27]), social cognitive theory [28], integrative model of behavioural prediction [29, 30], the capability, opportunity and motivation behavioural (COM-B) model [31] and social practices approach (SPA) [26].

The development of models and approaches such as COM-B and SPA have contributed to a shift in focus from individual attitudes (in theory of planned behaviour) to combining human agency with social structures [31, 32]. These approaches argue that a change in eating habits is not only governed by individual consumer attitudes and motivations, but also by the societal practice of daily eating routines. These practices are not fixed, and behaviour can change following shifts in competencies, perceptions and material aspects [33], all of which can be influenced by different types of policy interventions.

Several types of public policies and interventions can be used to steer food consumption, including (i) administrative policies such as directives, regulations and voluntary agreements; (ii) market-based policies such as taxes and subsidies; (iii) information-based policies such as labelling, education and information campaigns; and (iv) behavioural policies including nudges and rationing [34].

Information-based policies and interventions are the most commonly applied [34, 35]. They aim to enable the “right” consumer choice by raising consumer knowledge, awareness and competence to choose sustainably [35–37]. However, it has also been shown that such interventions alone fail to stimulate long-term sustainable change [36, 38]. Thus, a combination of policy interventions can be more effective (e.g. [39]). Administrative policy interventions can be regulations, laws or standards for public procurement via which governments can influence the decision-making of consumers and other food system actors to contribute to more sustainable outcomes. Administrative policies and interventions have proved to be successful, for example, in lowering unhealthy fat and sugar content in food [40]. Market-based policy interventions affect the prices of specific foods so that they better reflect true social costs [41]. Behavioural policies and interventions affect the relative availability and presentation of different foods to influence consumer behaviour and selection [42, 43].

#### Previous evidence syntheses

There are several relevant reviews on measures to steer sustainable food consumption, and specifically on policies for the promotion of dietary changes and reduction of overconsumption. However, they either focus solely on reducing consumption of animal-based foods [34, 44] or on interventions that aim to trigger consumer behavioural change but that does not measure environmental effect [22, 23, 35, 45]. Some focus on only one type of policy or policy interventions, such as digital behavioural interventions [45]. Others focus only on one type of outcome, such as climate change mitigation [46, 47], food waste reduction [46] or health [21, 34, 48]. Most previous systematic reviews also primarily capture interventions by private actors or do not explicitly discuss the role of government in promoting sustainable food consumption [45, 46]. Finally, some other relevant reviews do not apply systematic evidence synthesis methodology [12, 20, 22, 23, 49]. To meaningfully support public policy-making there is a need for a comprehensive synthesis that collates the best available evidence on all possible public policy interventions aimed at establishing more environmentally sustainable food consumption patterns.

#### Objective of the review

This systematic map aims to collate and describe available research evidence on public policy interventions that are either implemented or suggested for establishing more environmentally sustainable food consumption patterns. Specifically, this systematic map aims to answer:

What evidence exists on the effects of public policy interventions for achieving environmentally sustainable food consumption?

The question can be broken down into the following elements:

Setting(s): Any geographic or economic setting.

Intervention(s): Public policy interventions implemented by national or sub-national governments (or suggested to be implemented by e.g., researchers) with explicit aim to achieve more environmentally sustainable food consumption patterns. We use IEE’s [50] definition of a (public) policy intervention that is, any course of action, programme or activity taken or mandated primarily by national (and subnational) actors. As mentioned above, public policy interventions include regulations, market-based incentives, information schemes, the provision of infrastructure and similar. Moreover, they often address a variety of measures including technologies, processes, practices and behaviours to change food consumption patterns.

Outcome(s): Anticipated or actual change in any type of environmental outcomes of food production, or proxies thereof. Such proxies include e.g., change in meat or other animal-based food consumption, plant-based food consumption, consumption of food with high deforestation risk and consumption of environmentally certified products. Here we define sustainable food consumption as the use of goods and services that respond to basic needs and bring a better quality of life, while minimizing the use of natural resources, toxic materials and emissions of waste and pollutants over the life cycle, so as not to jeopardize the needs of future generations [10].

To inform the development of policies and policy interventions aimed at changing environmental effects from food consumption, the key outputs of this review will be as follows:

1. A detailed searchable database of relevant studies, including an overview of:
  - Proposed or existing policy interventions;
  - Measured effects of those interventions;
  - Any other relevant metadata such as bibliographic information, study location, type of study and similar
2. Visualisations of the evidence base including:
  - A series of ‘heat maps’ created by cross-tabulating two descriptors (e.g., policy type, “policy intervention type” and study type, etc.) to systematically identify knowledge clusters (i.e., subtopics that are well-represented in the evidence base) and knowledge gaps (i.e., subtopics that are un- or under-represented in the evidence base);
  - An evidence atlas i.e., interactive geographical map visualising the locations of the included studies (or authors’ affiliations).

This systematic map is a part of the project titled “*Towards a sustainable Swedish food system—a knowledge synthesis of environmental impacts and policy options*” funded by the Swedish Environmental Protection Agency. The work presented here has a global scope, but it is aimed at informing the Swedish context and is applicable to high-income countries. This focus is reflected in our stakeholder engagement strategy, grey literature searches and meta-data coding (see details below).

## Methods

The systematic map follows the guidelines and standards of the Collaboration for Environmental Evidence for evidence synthesis in environmental management [51] and it conforms to ROSES reporting standards (see Additional file 1).

### Stakeholder engagement

To assure the relevance of the review findings for stakeholders and better evidence uptake into policy and practice, a co-design process with continuous stakeholder input will be applied throughout the systematic mapping [52].

Key stakeholders for this study are food systems researchers and other types of researchers with an interest in food systems and sustainable food consumption, government actors at the national, regional and municipal level, civil society organisations and other actors along the food value chain. Apart from the expert and stakeholder group already associated with the project, which was set up at the project design stage, other stakeholders were identified via snowballing and systematic online searching as well as by using existing contacts of the review team, experts and stakeholder groups associated with the project.

Input to the systematic map design was solicited through a 2-stage process. First, we ran a workshop with key actors from the Swedish food system context to discuss the review scope and relevance of the systematic map for their work. Sixteen workshop attendants included representatives from the Stockholm Consumer Cooperative Society (1), the Swedish Co-operative Union (1), Swedish Consumer Agency (1), the National Food Agency (2), the Swedish Environmental Protection Agency (1, funder of the project), the Swedish Board of Agriculture (2), representatives for regions, cities and municipalities (6), food retail association (1) and a regional node for sustainable development and an innovative food system (Matlust, 1). Summary of provided feedback received during this process is available in Additional file 2.

Second, we collected comments to a draft version of the protocol, via an online survey form, administered to a broader group of Swedish and international stakeholders. The open consultation lasted 4,5 weeks, from the 12th of January to the 14th of February 2022. The stakeholders were asked for input on the scope, search strategy, and sources of grey literature. All the inputs were anonymous but stakeholders could leave their contact details for future project updates. The survey for feedback on the protocol was shared on the project website and via several relevant networks (for example TABLE, SLU Future Food, Mistra Food Futures, Mistra Sustainable Consumption) and social media (Twitter). We received 14 responses from researchers (5), policy-makers (2), research funders (1) and other actors along the food value chain (6). The protocol was updated following this process. A summary of provided feedback received during the open consultation process is available in Additional file 2 and the original survey form is in Additional file 3. Based on the stakeholder input we edited our search string and added more terms related to plant-based foods and vegetables, additional policy interventions (such as bans, prohibits, standards and mitigation) as well as additional environmental outcomes (such as biodiversity and land use). We also further described the categorisation of policy interventions and made additions to the list of specialist websites for grey literature searches.

At the end of the mapping process, we intend to discuss preliminary findings with the identified stakeholders to check for clarity of reporting and assure the relevance of the outputs.

### Search strategy

We will use a multi-pronged search strategy as detailed below. Searches will be conducted without any time limitations.

#### Bibliographic searches

We will conduct bibliographic searches using the following sources:

1. Scopus
2. Web of Science Core Collections (consisting of the following indexes: SCI-EXPANDED, SSCI, A&HCI, CPCI-S, CPCI-SSH, and ESCI)
3. The ProQuest Dissertation & Theses
4. Econlit
5. Applied Social Sciences Index & Abstracts (ASSIA)

Searches will be performed using English language search terms. Subscriptions from the Swedish University of Agricultural Sciences and Stockholm University will be used to access subscription services above (such as Web of Science).

#### Search strings

The search string will be composed of three substrings described in Table 1 combining setting (A), intervention (B) and outcome (C) terms as follows: A AND B AND C. Depending on search functionalities, the search string will be adapted to each bibliographic source (details are available in Additional file 4).

**Table 1:** Search substrings (shown as formatted for Web of Science)

A	(((food OR meal* OR diet OR eating) NEAR/2 (purchas* OR select* OR choice* OR reduc* OR choose OR decision OR consum* OR intake OR behav* OR habit*)) OR "product select*" OR "food products" OR menu OR "food environment" OR "dietary pattern*" OR catering OR ((beverage* OR grocery OR groceries OR fish OR seafood OR beef OR meat OR dairy OR milk OR vegetable* OR legume* OR “meat alternative” or “organic food” or “local food”) NEAR/2 (consum* OR choice* OR choose OR select* OR market OR demand* OR reduc*)))
B	(policy OR policies OR legislat* OR law* OR ((label* OR labl* OR certifi*) NEAR/2 (food OR ecol* OR sustainab* OR carbon OR climate)) OR ecolabl* OR ecolabel* OR eco-labl* OR eco-label* OR eco-certif* OR guideline* OR guidance OR incenti* OR intervent* OR nudg* OR subsid OR stimul* OR persua* OR “voluntary agreement*” OR roundtable* OR forc* OR innovat* OR directive* OR regulation OR regulations OR education OR ((plate* or serving-size or "serving size") NEAR/1 ration*) OR ((carbon OR consum* OR output OR environmental) NEAR/2 (tax* OR information OR standard* OR ban* OR prohibit* OR limit* OR sanction*)) OR "green criteria" OR "public procurement" OR "green public procurement")
C	("climate change" OR "climatic change" OR "global warming" OR "greenhouse gases" OR ghg OR "greenhouse effect" OR "greenhouse gas" OR "carbon emission*" OR "carbon footprint" OR "water footprint" OR “land use” OR “biodiver*loss*” OR ecosystem OR overfishing OR pollution OR "over fishing" OR deforest* OR (reduction* NEAR/2 emission*) OR (environment* NEAR/2 (impact* OR consequence* OR assess* OR evaluat* OR indicator* OR mitigat*)) OR "plant based food" OR "plant-based food" OR “planetary health diet” OR plant-forward OR "pro-environmental" OR "local food" OR "seasonal food" OR "eat less" OR flexitarian OR vegan OR vegetarian OR pescetarian OR "meat reduction" OR "beef reduction" OR (sustainab* NEAR/2 (consum* OR diet* OR food OR fisher*)))

Identification of the search terms shown in Table 1 below was based on the input from the subject experts, relevant literature and stakeholders. The search string was developed iteratively during a scoping phase (see Additional file 4).

#### Search engines

Searches in Google Scholar will be performed in English and main Nordic languages as per the skillset of the review team (Swedish, Danish and Norwegian) (see Additional file 4). We will use simplified sets of search strings, combining both intervention and outcome terms. The first 1000 search results will be extracted as citations using Publish or Perish software [53] and introduced into the duplication removal and screening workflow alongside records from bibliographic databases.

#### Specialist websites

Searches in specialist websites will be particularly important for capturing grey literature. Specifically, searches will be performed across a suite of relevant organisational websites (Table 2). The list of the relevant websites is being compiled with inputs from stakeholders. Each website will be hand‐searched for relevant publications using English and/or Nordic languages as appropriate.

**Table 2:** List of organisational websites including search languages

Website	Search language
1) IPCC, https://www.ipcc.ch/	English
2) FAO, http://www.fao.org/home/en/	English
3) UNEP, https://www.unep.org/	English
4) IFPRI, https://www.ifpri.org/	English
5) WHO, https://www.who.int/	English
6) OECD, https://www.oecd.org/	English
7) Table (previously the Food Climate Research Network) https://www.tabledebates.org/)	English
8) iPES FOOD, http://www.ipes-food.org/	English
9) Chatham House, https://www.chathamhouse.org/	English
10) WWF, https://wwf.panda.org/	English, Swedish, Norwegian, Danish
11) Swedish Board of Agriculture, https://www.government.se/government-agencies/swedish-board-of-agriculture/	Swedish
12) Norwegian Ministry of Agriculture and Food, https://www.regjeringen.no/en/dep/lmd/id627/	Norwegian, English
13) The Danish Ministry of Environment and Food, https://en.fvm.dk/the-ministry/	Danish
14) Finnish Food Authority, https://www.ruokavirasto.fi/sv/	Swedish
15) Swedish Environmental Protection Agency, https://www.swedishepa.se/	Swedish
16) Swedish Food Agency, https://www.livsmedelsverket.se/en	Swedish, English
17) The Swedish Consumer Agency, https://www.konsumentverket.se/	Swedish, English
18) Swedish Agency for Economic and Regional Growth, https://tillvaxtverket.se/	Swedish, English
19) Swedish Public Health Agency, https://www.folkhalsomyndigheten.se/	Swedish, English
20) Swedish Agency for Marine and Water Management, https://www.havochvatten.se	Swedish, English
21) European Union policies, https://ec.europa.eu/info/policies_en	English
22) Stockholm Resilience Centre https://www.stockholmresilience.org/	Swedish, English
23) Stockholm Environment Institute https://www.sei.org/	Swedish, English
24) Global Utmaning https://globalutmaning.se/	Swedish, English
25) Swedish public procurement agency https://www.upphandlingsmyndigheten.se/	Swedish, English
26) World resources institute https://www.wri.org	English
27) Hållbar livsmedelskedja https://hallbarlivsmedelskedja.se/	Swedish, English
28) Eat forum https://eatforum.org/	English

#### Additional searches

We will contact experts, the project’s reference group and stakeholders for relevant research (see Stakeholder engagement section) and calls for evidence will be issued on Twitter, LinkedIn, ResearchGate at minimum, including similar platforms and social networks. Finally, bibliographies of relevant reviews identified during the search process will be checked for relevant primary research literature.

#### Testing comprehensiveness of searches

During the scoping phase, search results were screened against a benchmark list of 38 articles with known relevance to the review. This was done to examine whether these searches were able to locate relevant articles (see Additional file 4). In cases where relevant articles from the benchmark list were not found with the search strategy, the search strings were examined to identify why articles were missed. Search strings were then adapted where relevant. The final search string captures all articles from the benchmark list except two (as these articles did not include mentions of food (consumption)-related terms in their titles, abstracts, or keywords).

#### Assembling a library of search results

Results of the bibliographic searches will be combined, and duplicates will be removed prior to screening. A library of search results will be assembled in EPPI-Reviewer [54].

### Article screening and study eligibility criteria

#### Screening strategy

The screening will be done by at least two reviewers and at two levels: at title and abstract (screened concurrently for efficiency) and at full text. Full texts of records with relevant abstracts will be retrieved, tracking those that cannot be located or accessed and reporting these in the final review. Retrieved records will then be screened at the full text, with each record being assessed by one experienced reviewer.

Prior to commencing screening, consistency checking will be performed on a subset of records at both title and abstract and full text levels. Specifically, up to 300 title and abstracts and 50 full text records will be independently screened by all reviewers (per round). The results of the consistency checking will then be compared among reviewers and all disagreements will be discussed in detail. Where the level of agreement among reviewers is low (below 80%), further consistency checking will be performed on an additional set of articles. This will be repeated until the consistency level reaches at least 80%.

Given that we expect a high number of search results we will use machine learning functionality available in the EPPI-reviewer to support the title and abstract screening process. We will combine the priority screening function with supervised classification. We will also use a subset of items screened by at least two reviewers as a training batch for building predictive modelling classifications. The final report will include specifications of the final model used for supervised classification (e.g., recall, accuracy).

We will provide a list of articles excluded at title and abstract, and at the full text, with reasons for exclusion in the final report. Reviewers who have also authored articles to be considered within the review will be excluded from decisions regarding the inclusion of their work.

#### Study eligibility criteria

The following criteria will be adopted for the inclusion of eligible studies:

Eligible settings: Private and public food consumption in any geographic or economic setting.

Eligible interventions: Public policy interventions with the explicit aim to achieve more environmentally sustainable food consumption patterns (implemented by national or sub-national governments or suggested). This includes policy interventions aimed at achieving change in consumption of e.g., meat, plant-based foods, deforestation-prone foods, or certified products. The intervention should have the end goal to directly or indirectly influence consumer choice towards more sustainable foods.

Specifically, we will include policy interventions with different governance styles (voluntary, mandatory, and collaborative) and that target information provision, the price of food or choice architecture. In Table 3 we provide examples of eligible policy interventions categorised according to the method applied in the systematic review by Temme et al. (2020) on policy interventions for reducing consumption of animal-sourced foods. This categorisation is just an example, and the list will be expanded and further categorised during the review process. Production-side interventions such as agricultural subsidies to promote more sustainable farming or interventions to restrict energy use in food production will not be included. Similarly, certification aimed at primarily changing production, and not primarily at promoting a more sustainable consumer choice, will not be included. Policy interventions that aim at the reduction of overconsumption (food eaten in excess or food wasted) will not be considered in this review.

**Table 3:** Examples of eligible policy interventions (to be expanded during the review process)

Policy type	Details	Governance style
Administrative	Laws	Mandatory
	Monitoring standards and sanctions	Voluntary, mandatory
	Standards for public procurement	Voluntary, mandatory
	Directives	Voluntary, mandatory
	Voluntary agreements	Voluntary
Market-based	Taxes, subsidies and changes to relative prices	Mandatory, collaborative
	Research funding	Collaborative
Information-based	Labelling/certification	Voluntary, mandatory
	Information campaigns	Voluntary, mandatory
	Marketing regulation	Mandatory
	Roundtables	Collaborative
	Capacity building	Collaborative
Behavioural	Nudging	Voluntary, mandatory
	Plate-, serving-size or rationing	Voluntary, mandatory
	Choice editing	Voluntary, mandatory

Eligible policy/policy intervention scales: Local (at universities, schools, hospitals and similar institutions), regional, municipality or national level.

Eligible target group of the policy/policy intervention: Food service sector, including consumers, retailers, food industry (marketing, food reformulation), restaurants, public procurement, cooperatives, supermarkets and similar. Policy interventions regulating the production side (e.g., regulation of pesticide use) or directed at primary producers are out of scope.

Eligible outcomes: Anticipated or actual change in any type of environmental impacts of food production, or proxies thereof including e.g., change in meat consumption, plant-based foods, consumption of deforestation prone foods or certified products. Studies that measure and/or discuss health outcomes only will not be included, although the interventions included in such studies, e.g., reduced overconsumption or consumption of red meat, might lead to environmental gains. Studies that do not explicitly measure environmental outcomes (directly or indirectly) will not be included.

Eligible study designs: Modelling, observational and experimental studies, theoretical and conceptual studies, studies with the quantitative, mixed method and qualitative data, primary and secondary research (including all types of reviews).

Eligible languages: English, Swedish, Danish and Norwegian (as per skillset of the review team).

Time frame: No limitations.

### Study validity assessment

The validity of studies will not be appraised as part of this systematic map, but we will extract information on a study design that can be used for and expanded during the critical appraisal in future systematic reviews based on this map [55].

### Data coding strategy

To assure repeatability of this stage, meta-data coding will be performed by multiple reviewers on a subset of up to 20 full texts, discussing all disagreements and clarifying coding scheme where needed.

If resources allow, we may contact authors by email with requests for missing information or clarifications. Whenever information was to be retrieved in other ways than directly from the document, this will be annotated and reported in the final review. Consultations with stakeholders and the reference and expert group will be used to develop the meta-data extraction protocol, but the following variables will be included (at minimum):

Bibliographic information, including publication details: list of authors, publication year, search source and publication type.

Study type, including modelling, observational and experimental studies, theoretical studies, reviews.

Research inquiry (for primary research): qualitative, quantitative, mixed.

Study location (and/or first author affiliation) at country level.

Policy and/or policy intervention type, including (but not limited to) administrative, market-based, information-based, behavioural, and similar (see Table 3 for examples above).

Level of the jurisdiction of policy/policy intervention levers, in terms of order of government likely responsible, such as local/municipal, provincial/regional/state/territorial, and national, and similar.

Actor(s) targeted: Consumers, retailers, food processors, traders, and similar.

Outcomes targeted: Any environmental outcomes targeted by the policy/policy intervention.

Outcomes measured, including (proxies for) environmental impacts.

Facilitators and barriers to reaching policy impact (if reported)

Relevant environmental policy objective: One or more Sweden’s 16 environmental quality objectives targeted by the policy/policy intervention and relevant Sustainable Development Goal(s).

Theoretical frameworks/approaches used in the studies: e.g., SPA.

Additionally, we also aim to record if studies have assessed conflicts/synergies between environmental objectives, indirect effects/spill-overs, distributional, legal, or political (e.g., acceptance) aspects of the policy intervention.

### Study mapping and presentation

The evidence base will be described narratively (using tables and graphs) and presented within a systematic map database, *i.e.*, a searchable spreadsheet containing codes and meta-data (as described in section *Data coding strategy*). We will produce heat maps to visualize knowledge clusters and knowledge gaps by cross-tabulating two variables (e.g., interventions and outcomes). The interactive visualizations of the mapping outputs will be made freely available on a project website.

The knowledge clusters identified by the systematic map will then be used to carry out a smaller number of in-depth policy reviews if deemed possible. The policy intervention selection will be made in dialogue with stakeholders and the reference group and focus on policy interventions where there is sufficient evidence to systematically assess the effectiveness and are deemed to be suitable for the Swedish policy context (e.g., policies implemented in other EU countries).

## Supplementary Information

**Additional file 1.** ROSES form for systematic map protocols.

**Additional file 2.** Summary of stakeholder input.

**Additional file 3.** Online survey form.

**Additional file 4.** Scoping exercise, search strategy and benchmark list.

## Data Availability

Not applicable.
