# Supplementary material for: What evidence exists on the effects of public policy interventions for achieving environmentally sustainable food consumption? A systematic map protocol
Source: Environ Evid. 2022 Apr 25;11:17. doi: 10.1186/s13750-022-00271-1 (PMC11378822; doi:10.1186/s13750-022-00271-1)
Supplement: Supplementary file 1 — Additional file 1. ROSES form for systematic map protocols. [file 13750_2022_271_MOESM1_ESM.pdf]

Section /	Topic	Description	Further	Checklist/Meta-	Author response	Comments
Title	Title	The title must indicate that it is a systematic map protocol, and	The title should	Meta-data	What evidence exists on the effects of public policy interventions for achieving environmentally sustainable food consumption patterns?	
Type of	Type of review	Select one of the following types	See CEE	Meta-data	systematic map	
Authors	Authors contacts	The full names, institutional		Checklist	Yes	
Abstract	Structured summary	Abstract must not exceed 350		Checklist	Yes	
Backgrou	Background	Describe the rationale for the	A theory of	Checklist	Yes	
Stakehol	Stakeholder engagement	The planned/actual role of		Checklist	Yes	
Objective	Objective	Describe the primary question and	The primary	Checklist	Yes	
	Definitions of the question components	Break down and summarise question key elements e.g. population, intervention(s)/exposure(s), comparator(s), and outcome(s).	For other question types see [4,5]	Meta-data	<u>Setting(s)</u>: Any geographic or economic setting<br><u>Intervention(s)</u>: Public policy interventions implemented by national or sub-national governments or suggested by e.g., researchers, with the explicit aim to achieve more environmentally sustainable food consumption patterns. Policy interventions include regulations, market-based incentives, information schemes, the provision of infrastructure and similar.<br><u>Outcome(s)</u>: Anticipated or actual change in any type of environmental outcomes of food production,	
Methods						
Searches	Search strategy		Details	Checklist	Yes	
	Search string	Provide Boolean-style full search string and state the platform for which the string is formatted (e.g. Web of Science format)		Meta-data	(((food or meal* or diet or eating) NEAR/2 (purchas* or select* or choice* or reduc* or choose or decision or consum* or intake or behav* or habit*)) or "product select*" or "food products" or menu or "food environment" or "dietary pattern*" or catering or ((beverage* or grocery or groceries or fish or seafood or beef or meat or dairy or milk or vegetable* or legume* or "meat alternative" or "organic food" or "local food") NEAR/2 (consum* or choice* or choose or select* or market or demand* OR reduc*))) AND (policy or policies or legislat* or law* or ((label* or labl* or certifi*) NEAR/2 (food or ecol* or sustainab* or carbon or climate)) or ecolabl* or ecolabel* or eco-labl* or eco-label* or eco-certif* or guideline* or guidance or incenti* or intervent* or nudg* or subsid* or stimul* or persua* or "voluntary agreement*" or roundtable* or forc* or innovat* or directive* or regulation or regulations or education or ((plate* or serving-size or "serving size") NEAR/1 ration*) or ((carbon or consum* or output or environmental) NEAR/2 (tax* or information or standard* or ban* or prohibit* or limit* or sanction*)) or "green criteria" or "green public procurement" or "public procurement") AND ("climate change" or "climatic change" or "global warming" or "greenhouse gases" or ghg or "greenhouse effect" or "greenhouse gas" or "carbon emission*" or "carbon footprint" or "water footprint" or "land use" or "biodiver*loss*" or ecosystem or overfishing or pollution or "over fishing" or deforest* or (reduction* NEAR/2 emission*) or (environment* NEAR/2 (impact* or consequence* or assess* or evaluat* or indicator* or mitigat*)) or "plant based food" or "plant-based food" or "planetary health diet" or plant-forward or "pro-environmental" or "local food" or "seasonal food" or "eat less" or overconsumption or overeating or flexitarian or vegan or vegetarian or pescetarian or "meat reduction" or "beef reduction" or (sustainab* NEAR/2 (consum* or diet* or food or fisher*)))	
	Languages –	List languages to be used in		Meta-data	English	
	Languages – grey	List languages to be used in		Meta-data	English, Swedish, Norwegian, Danish	
	Bibliographic databases	Provide the number of		Meta-data	5	
	Web – based search	Provide the number of web –		Meta-data	1	
	Organisational websites	Provide the number of		Meta-data	28	
	Estimating the	Describe the process by which the		Checklist	Yes	
	Search update	Describe any plans to update the	Optional. A	Checklist	n/a	
Article	Screening strategy	Describe the methodology for		Checklist	Yes	
	Consistency checking	Describe clearly the process for		Checklist	Yes	
	Inclusion criteria	Describe the inclusion criteria used		Checklist	Yes	
	Reasons for exclusion	State that you will provide a list of		Checklist	Yes	
Critical	Critical appraisal strategy	Describe here the method you	Optional	Checklist	Yes	
	Critical appraisal used in synthesis	Describe how the information from critical appraisal will be used in synthesis.	Optional	Checklist	No	Critical appraisal will not be conducted as such, see protocol for details
	Consistency checking	Describe how repeatability of	Optional	Checklist	No	
Data	Meta-data extraction	Describe the method for meta-		Checklist	Yes	
Data	Narrative synthesis	Describe methods to be used for	Vote-	Checklist	Yes	
	Knowledge gap and	Describe the methods to be used		Checklist	Yes	
	Demonstrating	Describe the role of systematic	Reviewers	Checklist	Yes	
Declaratio	Competing interests	Describe of any financial or non-		Checklist	Yes	

Referenc

[1] James, K.L., Randall, N.P. and Haddaway, N.R., 2016. A methodology for systematic mapping in environmental sciences. Environmental Evidence, 5(1), p.7.

[2] Bayliss, H.R., Haddaway, N.R., Eales, J., Frampton, G.K. and James, K.L., 2016. Updating and amending systematic reviews and systematic maps in environmental management. Environmental Evidence, 5(1), p.20.

[3] Haddaway, N.R., Kohl, C., da Silva, N.R., Schiemann, J., Spök, A., Stewart, R., Sweet, J.B. and Wilhelm, R., 2017. A framework for stakeholder engagement during systematic reviews and maps in environmental management. *Environmental Evidence* , *6* (1), p.11.

[4] Collaboration for Environmental Evidence. 2018. Guidelines and Standards for Evidence synthesis in Environmental Management. Version 5.0. www.environmentalevidence.org/information-for-authors.

[5] Leeds Institute of Health Sciences. https://medhealth.leeds.ac.uk/info/639/information_specialists/1500/search_concept_tools. Accessed 12/11/2017.
