# Supplementary material for: What evidence exists on the effects of public policy interventions for achieving environmentally sustainable food consumption? A systematic map protocol
Source: Environ Evid. 2022 Apr 25;11:17. doi: 10.1186/s13750-022-00271-1 (PMC11378822; doi:10.1186/s13750-022-00271-1)
Supplement: Supplementary file 2 — Additional file 2. Summary of stakeholder input. [file 13750_2022_271_MOESM2_ESM.docx]

# Additional file 2: Summary of stakeholder input

This additional file includes following:

- Table 1 summarises written feedback from the stakeholder expert group, including our responses.
- Table 2 (part1) and 3 (part 2) provide an overview of the comments received via the online survey during the open consultation process and it includes our responses. We have removed survey answers that refer to the background information of respondents. The original survey form is in Additional file 4.
- Table 4 summarises the feedback on the search string including our responses. The comments are from the expert group and the stakeholders that participated in the online consultation process.

# Table 1: Feedback from the stakeholder expert group

| **Comments** | **Response** |
| --- | --- |
| The setting in section 2 is in any geographic or economic, but in and part A in 3.2.2 the words seem to imply that A is “food consumption” in general? Rather than the geographic or economic setting? I wasn’t sure if they were supposed to correspond. | Section A is simply a food consumption string. The entire search string is compiled to encompass any context as per the eligibility criteria specified in section 3.3. |
| I also wasn’t sure if the plant-based/seasonal/local etc. is in the outcome – are those then the proxies for the environmental outcomes. Maybe a table earlier on with all the environmental outcomes and then all the proxies would be good to understand what is included. | We would like to remain broad and open about all potential environmental outcomes and therefore we do not list all of them explicitly in the search string as our scoping search proved that this generates too many records that are on specific topics only related to the environmental outcomes, not on our scope.  The same argument is applied to the specification of proxies; we will include studies that can present a proxy to assessing change in environmental impact but which these proxies are more specifically we would like to keep broad as to include as many studies assessing impact/proxies of impact as possible |
| For the organization of the public policy measures, a framework for the instrument type could be adopted. Here are a few references, that I thought could perhaps be of interest on frameworks used to categorize SC policy interventions into instrument types: (Dawkins et al., 2019; Grubb et al., 2020; Kiss et al., 2018; Reisch et al., 2013; Schroeder, 2014; Sibbing et al., 2019; Wolff and Schönherr, 2011). Some more general ones: (Hood, 1983; Howlett, 2011; Schneider and Ingram, 1990). And a Swedish EPA references: (SEPA, 2012). | Thank you for this valuable feedback. We will wait with the final categorisation of the policies until we have collected the data. Table 3 is providing an example of policy interventions and how these can be categorised following a study by Temme et al. (2020) on policies for decreasing animal sourced food consumption. We will revisit this study as well as others (including those mentioned here) to identify the best way to categorise the policy interventions we identify in this mapping.  We have further clarified that the categorisation and table 3 are just examples of policy interventions and a way of categorising them |
| For the suggested rather than implemented policies, a couple of thoughts, firstly, will these come from government vision documents/strategies plans etc. or just the academic literature, or they will be excluded as not studies as such? Secondly, perhaps it could be useful to make distinct in the review and the searchable database (e.g., under measured effects) whether the studies evaluated the outcome of the public policy invention or not and then how the evaluation was performed. See Dawkins et al. (2019) we found limited evaluations and when they did they might have been quite narrow – or not evaluating both process and outcome. There was also a distinction between qualitative v quantitative evaluation, with some studies focusing on one or the other mainly. | We will search academic literature as well as “grey literature”, including government webpages.  We will also collect data on how studies measure and evaluate the outcome of the public policy/intervention as this is an eligibility criterion for inclusion in our study.  We will study this subject deeper and try to collect as much data as possible on how the effect of policies/interventions are measured. |
| Define environmental outcomes early on in the text, which will then determine the search string. E.g., in the outcomes section under part 2 (objectives of the review) you have some proxies, but not the actual measures. Might be good to also list what any type of environmental outcome includes first. | We appreciate this comment but please see our earlier response. |
| In eligible interventions in 3.3.2 the end goal is to directly or indirectly influence consumer choice –could be broadened to deliver sustainable food consumption, as the choice might stay the same. Governance styles is also mentioned here (like voluntary, mandatory etc.) perhaps discuss this a bit more higher up in relation to the classification of instruments e.g. see (Palm et al., 2019). | This has been changed in the protocol to add that the end goal is to directly or indirectly influence consumer choice towards more sustainable foods. |

# Table 2: Feedback from stakeholders provided during public consultation process via the online survey (part 1)

| **Respondent ID** | **Our study focus on consumption-side policies for a more environmentally sustainable food**  **Consumption.**  **We are including studies that are looking at anticipated or actual change in any type of environmental**  **outcomes of food production. For example:**  **- change in meat or other animal-based food consumption'**  **- plant-based food consumption**  **- consumption of food with high deforestation-risk**  **- consumption of environmentally certified products.**  **- studies that measure changes in, for example, actual carbon or water footprints**  **Are there any other outcomes that we should include in our study?** | **Are there other search terms (than provided in the search string) that you think are missing in our search string? Please specify.** | **We are focusing on public policies in this systematic mapping. Examples of categories and policies are**  **outlined in the table below. The categorisation will be based on data input but this provides a general**  **idea of what the study aims to include**  **Are there any policy categories or policy interventions that you see are missing or that would not be covered based on the examples provided in the table?** | **Do you have any other (specific or general) reflections that you would like to share with us? Please specify.** | **Response** |
| --- | --- | --- | --- | --- | --- |
| 1 | Including organically certified produce, ethically marked produce, amount of fruit and vegetables consumed, type of fruit and vegetables consumed | no |  |  | Production side policy interventions falls outside the scope of our study |
| 2 | Transition from high greenhouse gas fertiliser manufacture to zero carbon manufacture is an important risk. Deforestation is complex since in much of Europe the climax vegetation is woodland and our wealth creation has led to reduction while we expect others to "do as we say and not do as we have done". It is particularly important to understand the implications for European consumption that can directly impact on others in the world pushing the price of food out of reach (sometimes with systems such as organic without a consistent science basis). | Implications for others in the global economy | Animal welfare, external implications for antibiotic use, pesticide resistance, GM development | Europe tends to be introspective in its food policy and appears reluctant to allow consumers to make food purchase decisions based on their own informed view. It is easy to impose our view on others even when just our ethical choice. | The comments on the search string and protocol falls out of the scope of our study as we do not focus on production side interventions or target groups affected by consumers |
| 3 | (agro)biodiversity, soil quality, pesticide and fertilizer use, packaging-related pollution | x | x | x | Food environment, eutrophication and water footprint are already included in the search string |
| 4 | Ett förslag är att vidga studien till att inte bara inkludera avskogning utan också påverkan på biologisk mångfald i bredare bemärkelse.  A suggestion is to widen the study to include biodiversity and not only deforestation. | Nej | Nej | Nej | Biodiversity loss has been added to the search string. See also table 4 for amendments to the search string |
| 5 | -consumption based on locally/nationally available foods. -consumption based on foods available per season. | planetary health/planetary health diet, plant-forward |  |  | Planetary health diet and plant-forward has been added to the search string.  Locally available foods and seasonal foods are included if they are measured/assessed as proxies for change in environmental impact |
| 6 | other types of footprints could be relevant to include such as nitrogen, phosphorus, eutrophication, or land (with a focus on biodiversity). The issue with environmentally certified foods is that they offer a great way to combine metrics but often have a bias towards what is easily measured and may not be good on all aspects (e.g., organic agriculture and nutrient management has some issues). | I am unclear here if you only care about Nordic focus or not. It seems to be a real systematic protocol to go global, but then for the specific greylit of course one needs to go with languages one knows. Later in the screening part I would suggest adding something that allows to bin for the Nordic context. In A If you are adding specific things like meat should you also add things like vegetables, legumes, meat alternatives, healthy, local, organic? I am just trying to think that could increase the types of studies that might have looked at increased uptake of ‘good foods’ (and not just decrease of high environmental cost items. I can see some of this in is list C, but depending on how you are combining things maybe some key words need to be in multiple locations? In B Are you going to add some words from the types of public policy intervention types you have already identified? seems like you have most of them (e.g., nudg) but I would do a double check in case there are a few more words that tend to come up in those review articles (thinking that environmental psychology might have a few go to terms). In C I’ll add my plug-in for nitrogen, phosphorus and eutrophication. Also maybe soil and water quality. There are not so many studies on this but I know of a few with nitrogen footprints that might fit in to the scope of the study. I also wonder if you want ot add something on trade given you also have local food here. | Here again is a bit of a bias suggestion as I will soon do research on it, but there could be some interesting things around participatory actions to change food choices (so I think it fits under behavioural but it has to do with cooking classes, gardening, or in general physically engaging with food differently). This might mean some different key words or maybe you just exclude it if its not at the right scale. | If the aim is really about the actual enviro impact, then local vs global supply chains and data gathering will matter in what exists in terms of doing that. What about studies that affect other parts of society that in turn affect consumption behaviours? So thinking of how food stamps work, or how other social benefits are given out? (so we have free school lunches here which gives power of some of it but that isn’t the point of school lunches). You give examples of some of your exclusions but I wonder if it will need to be a dynamic type of binning for the screening because of what is out there and how studies are perhaps siloed in certain fields. | See comments in in table 4 below on amendments on the search string |
| 7 | I think you should as well be looking at near produced products and the consequences of shorter freight distance. | I think it sounds good. | No other that I can think off. |  | We are looking at locally produced food in the current search string. If studies have shorter freight distance, and this is used, for example, as an informative nudge for consumers, such study will be included within our scope. |
| 8 | Be aware of risk when transferring results from one area of the world to another. | increase or decrease in agricultural products, food products or other. | don't know | Try to include the effects of changes on primary production. | Primary production is out of scope for this review |
| 9 |  |  |  |  |  |
| 10 | Economic sustainability for the primary production, Effects of National self-sufficiency | Nutritional-aspects if you should compare different types of foods (ex milk is more nutritional then water and soda), different groups in the population have different demands if you look at the nutritional aspect - how does that affect policies, nudging that are more likely to be general. |  |  | Nutritional aspects or primary production are out of scope for this review |
| 11 | The question of free seeds, local seed production and regulations concerning seeds. | No | No | People should be encouraged to grow their own food, nothing is as good as consumption from your back yard. | Primary production are out of scope for this review |
| 12 | What food is produced sustainably and whhat should be don to produce all food by sustainable methods? | Sustainable production of food. | This should be done in the first place for long term sustainable food production. inable foo | Instead of study use time and money for creating several type of toilets without water to make it possible for all people to collect toilet waste in hygienic and easy to use method. Then we need local high-tech biogas plants to better use of bioenergy and plant nutrients without polluting environment. | Not our focus. We do not focus on primary production |
| 13 | How the food is produced: Det är väldigt relevant för hållbar konsumtion om köttet är "industriproducerat" (tänk brasilien) eller regenerativt producerat (där det binder betydligt mer växthusgas än vad som produceras. Detta gäller även plant-based produktion. Där även ekomärkta produkter har en värre inverkan på jorden än konventionell regenerativ spannmålsproduktion. Viktigt att inte dra alla över samm kam.v  It is very relevant for sustainable consumption if the meat is “industrially produced”(think Brazil) or produced in regenerative production (where it can hold much more GHG emissions than what is produced). This is also valid for plant-based production. Where even eco-labeled products have a more negative and severe impact on the earth than conventional regenerative cereal production. It is important not to treat/judge every producer in the same way | regenerative food production, meat and plantbased | Prissättning: bra val ska gynnas och göras billigare med dåliga vals pengar/("straff-")skatt etc. Labelling: Även negativ märkning skulle behövas för att uttrycka att det som köps påverkar miljö och klimat negativt (som med cigaretter)  Price setting: ”good choices should be promoted and be made cheaper with the money that are payed for the “bad choices”/”(punishment-“) taxation etc. Labelling: Even negative labelling is needed to express that which impacts environment and climate negatively ( as with cigarettes) |  | We cover the suggestions regarding meat and plant-based with our current search string. We will also include taxation and labelling and other market regulations in our scope. Production side policies (like promoting regenerative production) and interventions are, however, out of the scope of this review. |
| 14 | Organic foods (not necessarily the same as environmentally certified). The issue with fish/overfishing might be included somehow in the indicators above, but could be a targeted impact (biodiversity). | mitigation | Maybe something related to coalitions (government and industry) which may not necessarily be a policy, but can move things in a certain direction | The search is very large/broad. Might make sense to split it up into several different searches to find relevancy. There are also no 'exclusion' criteria. That might help to reduce the 'fuzz' from different types of articles you don't want. | Mitigation added and biodiversity loss are added to the search string. See also table 3 below for amendments to the search string.  We have exclusion criteria, called “eligible criteria” described in section 3.3.2 in the protocol. |

# Table 3: Feedback from stakeholders provided during public consultation process via the online survey (part 2).

| **Respondent ID** | **We identified several sources of grey literature such as the following:**  **• at the webpages of IPCC, FAO, UNEP, IFPRI, WHO, OECD, Table, iPES FOOD, WWF**  **• The Danish Ministry of Environment and food, the Finnish Food Authority and the**  **Norwegian ministry of Agriculture and Food**  **• The Swedish Board of Agriculture, Food Agency, Environmental Protection Agency, Consumer**  **Agency, Health Agency, Agency for Marine and Water Management and Agency for Economic and**  **Regional Growth**  **• The European Union website for EU policies**  **Is there anything missing from the list of grey literature search webpages?** | **What articles should we not miss in this review?** | **Do you find that these statements correspond to your interests or do you have any concerns about the set study scope?** | **Comment/ Amendment** |
| --- | --- | --- | --- | --- |
| 1 |  |  | yes | No suggestions were made |
| 2 |  |  | Clearly important but potentially fails to separate environmentally sound production from production that allows space for environmental enhancement. There are also a number of conflicts for example where high welfare standards have higher greenhouse gas implications. | We do not focus on production and production-side interventions so this is outside our scope |
| 3 | HLPE reports | x | to what extent are you including policies that affect the supply side and food environments that promote sustainable consumption patterns? | We do not focus on production and production-side interventions so this is outside our scope.  Food environment is included in our search string and researchers that study this as a policy intervention to change consumption patterns towards more  sustainable food consumption will be included in the mapping |
| 4 | Nej | No suggestions | Reasonable limitations | No suggestions were made |
| 5 | https://hallbarlivsmedelskedja.se/ https://eatforum.org/ |  |  | Added to the list of webpages for grey literature search |
| 6 | Maybe Word Resource Institute | work done with the PRINCE dataset looking at the environmental impact of Swedish consumption. Not sure how much of the policy side they looked at but seems relevant to include. | The focus is good. I think linking all the way to actual environmental impact is great, but maybe difficult to find in the literature | World resources institute added to the list of grey literature web pages to search. We will also include work from the PRINCE project in our mapping. |
| 7 |  |  | I think it sounds good. | No suggestions were made |
| 8 | don't know | don't know | Risk of too narrow focus area. | No suggestions were made. We have a broad focus but our eligibility criteria on outcome, where we only include studies that measure a change/proxy of change in environmental impact will narrow the scope. |
| 9 |  |  |  |  |
| 10 |  |  | It correspond | No suggestions were made |
| 11 | No | I have no suggestions | I feel it is relevant to take part in this survey. | No suggestions were made |
| 12 | Why not www.biotransform.eu where are som suggestions hon to create sustainable farming. | All about soil degradation, unsustainable management of plant nutrients in systems for waste and wastewater management. Less than 2% of plant nutrients delivered to cities return to cultivated soils. See report from EllenMacArthur foundation 2019. - 98 % pollute air and water! | No. Hon you can do research on "more environmentally sustainable food consumption" Wien moster of food is produced by unsustainable cultivation methods? | We do not focus on sustainable farming as this falls outside our scope. |
| 13 | En liten, men så fin rapport: https://regenerationinternational.org/2021/03/08/best-practices-how-regenerative-organic-agriculture-and-land-use-can-reverse-global-warming/ | Laws, labelling, price | Ja delvis. Det är ju av vikt även policys på riksplanet vad som uppmuntras vid produktionen. T.ex underlätta och uppmuntra matproducenter att gå över till agroforestry/regenerativa odlings/betessystem. Det skulle jag säga är den viktigaste frågan för en hållbar framtida livsmedelsproduktion. Efter det så behövs märkning och dra ned det till 3 st så att det är enkelt att välja rätt: 1 Svenskt, 2 Eko 3 Regenerativ produktion.  Yes partly. It is important, that policies at national level are encouraged at production. For example by encouraging and facilitating the transition towards agroforestry/regenerative farming/grazing systems. I would say that is the most important question for sustainable food production in the future.  After that, labelling is neaded. Make it three labels so it is easy to make the right choice: 1) swedish, 2) ecological, 3) regenerative production | The suggested areas for articles that we should not be missed will be included in our mapping based on our search string.  Production-side policies are not included within the scope of this study |
| 14 | Swedish Public Procurement Agency. | Hard to say what you will miss. | Not all consumption based policies may be aimed directly at food (but food may be intrinsically covered) | The Swedish public procurement agency is added to our grey literature list of websites.  We agree that not all consumption-based policies focus on food consumption. 15We will consult reviews in other areas to discuss and frame our findings to a wider scope. |

# Table 4: Summary feedback received on the search string collected from all stakeholders (including the expert group and during open consultation process)

| Feedback | Responses and adjustments made to the string |
| --- | --- |
| Calor* intake is included, but no other health variables. Perhaps this could be removed, or expanded?  Could add standards next to guideline and guidance (B) | We have removed calor* intake. We still have intake with food, meal or diet in the string.  We have added standards next to guidelines |
| There is also reduc* in here, but should that be as part C, an outcome? | No – this means reduce as a synonym to food consumption. There is also emissions reduction in C |
| Could also add bans/prohibits and limits. (B) | We have added ban, prohibit* and limit* with a bracket NEAR/2 carbon, consumption, output or environment |
| increase or decrease in agricultural products, food products or other. (A) | Not applicable in our framing |
| Could add standards next to guideline and guidance (B) | We have added this to the search string. |
| Mitigation (C) | We have added this to the search string. |
| Maybe incenti* with f and v. (B) | We have changed this in the search string. |
| regenerativ food production, meat and plantbased (A) | Not applicable in our framing |
| Sustainable production of food. | Not applicable in our framing |
| Nutrional-aspects if you should compare different types of foods (ex milk is more nutrional then water and soda), different groups in the population have diferent demands if you look at the nutrional aspect - how does that affect policies, nuddging that are more likely to be general.. (A) | Not applicable in our framing |
| Subsidies (B) | We have added this to the search string. |
| I’m not sure how it would influence the string exactly, but should tax and public procurement come higher up next to legislat* or law*? | This does not make any difference to the search results |
| vegetables, legumes, meat alternatives, healthy, local, organic (A) | We have added this to the search string in a bracket connected to consume, choice etc. |
| Some foods are mentioned explicitly – like beef and fish, but not others. Perhaps include a justification for this (e.g., is it based on known high env impact of those products), but why not pork or chicken for example. (A) | We have added this to the search string in a bracket connected to consume, choice etc. |
| Here there are also some specific foods like beef reduction, but not others, perhaps this could be broader. (C) | We have added this to the search string in a bracket connected to consume, choice etc. |
| Are you going to add some words from the types of public policy intervention types you have already identified? seems like you have most of them (e.g., nudg) but I would do a double check in case there are a few more words that tend to come up in those review articles (thinking that environmental psychology might have a few go to terms). (B) | This has been added in accordance with the table in the protocol |
| In C I’ll add my plugg in for nitrogen, phosphorus and eutrophication. Also maybe soil and water quality (C) | Nitrogen and phosphorous are captured in eutrophication and eutrophication is already included. |
| I also wonder if you want ot add something on trade given you also have local food here. (C) | Trade is not included in our framing. |
| could have ‘biodiversity footprint’/ ‘land footprint’ / ‘chemical footprint’ / pesticides etc. in the search string. Or biodiversity loss (as in key words?). (C) | Biodiversity loss and land use is added to the search string |
| In B will it be possible to distinguish between public policy and voluntary efforts – especially for labelling, they might not be driven by public policy, but a voluntary sector effort. (B) | This is something that we will address when we analyse our results. |
| One more general idea for setting would be to see whether the string captures specific government related areas where they could have influence via public provision of services like school/preschool/elderly meals, hospital food etc.? (but I’m not sure if/how it might fit into the string). (A) | This is specified in the search string as public procurement, catering, meal etc. We also have studies in the benchmark list with such formulation to ensure they are captured |
| you will not find many of the interventions/policies listed in Table 3 using the present search strings. I suggest you update search strings to include the words present in Table 3. | We have added words to capture the table in the protocol. Most were already included but we have added a few more. |
