# Supplementary material for: What evidence exists on the effects of public policy interventions for achieving environmentally sustainable food consumption? A systematic map protocol
Source: Environ Evid. 2022 Apr 25;11:17. doi: 10.1186/s13750-022-00271-1 (PMC11378822; doi:10.1186/s13750-022-00271-1)
Supplement: Supplementary file 3 — Additional file 3. Online survey form. [file 13750_2022_271_MOESM3_ESM.pdf]

Effects of public policy interventions for sustainable food consumption

This survey is intended to collect your comments on a methodological plan (protocol) for a systematic review on the effects of public policy interventions for sustainable food consumption and by filling out the survey you will help the research project to define correct barriers and collect the most relevant data.

The current global food system is causing considerably environmental harm. To increase environmental sustainability in food systems, production improvements are needed as well as changes in consumption patterns. To stimulate such changes, targeted public policy interventions are key. While there is extensive research devoted to exploring how more environmentally sustainable food consumption can be promoted, this knowledge is scattered across different sources. There is a need for a robust and comprehensive mapping and synthesis of existing relevant research and better brokering of scientific knowledge to policy and practice.

To answer above mentioned synthesis gap, we will conduct a systematic mapping of the literature to collate and describe available research evidence on public policies (either implemented or suggested) for establishing more environmentally sustainable food consumption patterns. Specifically, this systematic map will answer: What evidence exists globally on the effects of public policy interventions for achieving environmentally sustainable food consumption patterns?

We have drafted a protocol that outlines in detail our methodological plan for the systematic map. To give potential users of the map the opportunity to influence our work at an early stage, we have designed this survey. Your feedback will help us refine the protocol, and it will influence the scope and comprehensiveness of the map. At this stage, we are seeking input regarding:

• That we have identified the right study focus and aims to capture the right type of studies
• That our search string captures key literature
• That we include the all relevant policies in our study
• Where to find non-scientific research to include

The protocol can be downloaded at this website: https://osf.io/fj5um/ (https://osf.io/fj5um/) | Effects of public policy interventions for sustainable food consumption. While we encourage you to have a look at the protocol, it is perfectly possible to take the survey without reading the protocol.

This survey form will be open for your feedback until February 14th, 2021. Answering our questions will take only about 5 minutes. Feel free to share the survey and protocol within your networks.

This survey is part of the project "Towards a sustainable Swedish food system - a systematic mapping of literature on environmental impacts and policy options" which is funded by the Swedish Environmental Protection Agency. Please note that we will not collect your personal information. For any questions or concerns, please get in touch with Ylva Ran (ylva.ran@slu.se (mailto:ylva.ran@slu.se)).

We thank you for your time and valuable feedback!

1

What is your background?

- ○ Research with a focus on consumption
- ○ Research with a focus on food systems
- ○ Research other
- ○ Research funder
- ○ Technology development and commercialisation
- ○ Policy- and decision-makers for food systems at municipal level
- ○ Policy- and decision-maker for food systems at regional level
- ○ Policy- and decision-maker for food systems at national level
- ○ Agricultural/food production or other position in the food system
- ○ Non-governmental organization (NGO)
- ○ [ ] Other

Study focus

Our study focus on consumption-side policies for a more environmentally sustainable food consumption, We will focus on public policies, that is, policies that can be implemented and/or developed by national, regional or local governments.

We will not focus on studies with a primary interest to reduce food waste as this topic is covered by other systematic literature reviews.

We will not focus on studies with a primary focus to improve health

2

Do you find that these statements correspond to your interests or do you have any concerns about the set study scope?

Relevant impact

We are including studies that are looking at anticipated or actual change in any type of environmental outcomes of food production. For example:

- change in meat or other animal-based food consumption'
- plant-based food consumption
- consumption of food with high deforestation-risk
- consumption of environmentally certified products.
- studies that measure changes in, for example, actual carbon or water footprints

3

Are there any other outcomes that we should include in our study?

Search strategy

Your answers in this section will inform the search strategy described in section 3.2.2 of the protocol. The search strategy affects the comprehensiveness of our review.

So far, we have identified the search terms illustrated below to find relevant literature. The first sub-string (A) aims to capture studies on food consumption or equivalent words to describe food consumption. Sub-string B focus on policies or other types of word that describe policies and policy instrument. The third search string (C) focus on the environmental impact associated with policies for food consumption. The idea is to combine search terms as follows: A AND B AND C.

A Food consumption	• Food consumption/purchase/selection/choice/decision/intake<br>• Meal or diet choice/selection/purchase/choosing/decision/habit/intake<br>• Eating habit/behaviour<br>• Product selection, food products, menu, food environment<br>• Calorie intake, dietary pattern<br>• Catering<br>• Beverage - consumption/choice/selection/reduction/market or demand<br>• Grocery or fish or seafood or beef or meat or dairy or milk consumption/choice/selection/reduction/market or demand
B Policy	• Policies legislation, guidelines, guidance, interventions, incentives<br>• Nudges, stimulations, persuasions, forces, innovations<br>• Food or ecological or carbon or climate or sustainability labels/certification<br>• Carbon or consumption or output or environmental tax/information<br>• Green criteria, green public procurement
Environmental impact	• Climate change, global warming, greenhouse gas- and carbon emissions<br>• Carbon footprint, water footprint<br>• Ecosystem, overfishing, pollution, deforestation<br>• Emission reduction<br>• Environmental impact/consequence/assessment/evaluation/indicator<br>• Plant-based food, seasonal food, local food, flexitarian, vegan, vegetarian, pescetarian<br>• Eat less, overconsumption, overeating, meat reduction, beef reduction<br>• Sustainable consumption/deit/food/fisheries

4

Are there other search terms that you think are missing in our search string? Please specify.

Policy type	Details
**Administrative**	Laws
	Monitoring standards and sanctions
	Standards for public procurement
	Directives
	Voluntary agreements
**Market-based**	Taxes, subsidies and changes to relative prices
	Research funding
**Information-based**	Labelling/certification
	Information campaigns
	Marketing regulation
	Roundtables
	Capacity building
**Behavioural**	Nudging
	Plate-, serving-size or rationing
	Choice editing

Public policies

We are focusing on public policies in this systematic mapping. Examples of categories and policies are outlined in the table below. The categorisation will be based on data input but this provides a general idea of what the study aims to include

5

Are there any policy categories or policy interventions that you see are missing or that would not be covered based on the examples provided in the table?

6

What articles should we not miss in this review?

Inclusion of grey literature

Our study will also look at grey literature such as reports, theses, datasets and similar literature that is not published in scientific journals.

We identified several sources of grey literature such as the following:
• at the webpages of IPCC, FAO, UNEP, IFPRI, WHO, OECD, Table, iPES FOOD, WWF
• The Danish Ministry of Environment and food, the Finnish Food Authority and the Norwegian ministry of Agriculture and Food
• The Swedish Board of Agriculture, Food Agency, Environmental Protection Agency, Consumer Agency, Health Agency, Agency for Marine and Water Management and Agency for Economic and Regional Growth
• The European Union website for EU policies

7

Is there anything missing from the list?

8

Do you have any other (specific or general) reflections that you would like to share with us? Please specify.

9

If you would like to be informed about the progress of the review please get in touch with us. You can email us at ylva.ran@slu,se. You may also leave your email address below.

This content is neither created nor endorsed by Microsoft. The data you submit will be sent to the form owner.

Microsoft Forms
